# Disease transmission and mass gatherings: a case study on meningococcal infection during Hajj

**DOI:** 10.1186/s12879-022-07234-4

**Published:** 2022-03-22

**Authors:** Laurent Coudeville, Amine Amiche, Ashrafur Rahman, Julien Arino, Biao Tang, Ombeline Jollivet, Alp Dogu, Edward Thommes, Jianhong Wu

**Affiliations:** 1 Sanofi Pasteur, Lyon, France; 2 Sanofi Pasteur, Dubai, UAE; 3 Oakland University, Rochester, USA; 4 University of Manitoba, Winnipeg, Canada; 5 York University, Toronto, Canada; 6 Sanofi Pasteur, Toronto, Canada

**Keywords:** *Neisseria meningitis*, transmission dynamics, Mass gathering, Vaccine, Mathematical model

## Abstract

**Background:**

Mass gatherings can not only trigger major outbreaks on-site but also facilitate global spread of infectious pathogens. Hajj is one of the largest mass gathering events worldwide where over two million pilgrims from all over the world gather annually creating intense congestion.

**Methods:**

We developed a meta-population model to represent the transmission dynamics of *Neisseria meningitidis* and the impact of Hajj pilgrimage on the risk of invasive meningococcal disease (IMD) for pilgrims population, local population at the Hajj site and country of origin of Hajj pilgrims. This model was calibrated using data on IMD over 17 years (1995–2011) and further used to simulate potential changes in vaccine policy and endemic conditions.

**Results:**

The effect of increased density of contacts during Hajj was estimated to generate a 78-fold increase in disease transmission that impacts not only pilgrims but also the local population. Quadrivalent ACWY vaccination was found to be very effective in reducing the risk of outbreak during Hajj. Hajj has more limited impact on IMD transmission and exportation in the pilgrim countries of origin, although not negligible given the size of the population considered.

**Conclusion:**

The analysis performed highlighted the amplifying effect of mass gathering on *N. meningitidis* transmission and confirm vaccination as a very effective preventive measure to mitigate outbreak risks.

**Supplementary Information:**

The online version contains supplementary material available at 10.1186/s12879-022-07234-4.

## Background

Mass gatherings (MGs) are defined by the World Health Organization as concentrations of people at a specific location for a specific purpose, over a set period of time with the potential to strain local resources [1]. MGs come in many different forms but can be grouped into three main categories: social events (concerts, festivals, etc.), sport events (FIFA World Cup, Olympic Games, etc.) and religious events (Kumbh Mela, Hajj, etc.). Because of increased global mobility, MGs have become both more frequent and more attended.

MGs put a strain on the resources of the location hosting these events, from transporting and housing gatherers to crowd control and policing. From the perspective of health, MGs pose a variety of significant public health threats, so much so that in 2011, the Jeddah declaration on Mass Gatherings Health laid out principles organising the field of mass gathering medicine [2]. Health issues stemming from MGs typically involve increased risks of injuries and spread of communicable diseases between participants and the local population [3–5]. While the initial focus of the medical community was on dealing with issues arising in the host location, the problem evolved in recent years to include global health aspects [6, 7]. Two issues are of particular concern in this context. First, participants to MGs often originate from a variety of locations with different health systems, varying susceptibility, and immunity to pathogens. Therefore, one can ask what is the risk of infectious disease outbreak due to the MG event and how can this risk be mitigated? Second, if an outbreak occurs at the MG site, what is the risk that participants returning to their homes spread the infection to a previously unaffected region?

Here, we focus on the first of these questions and take transmission of invasive meningococcal disease (IMD) during Hajj as a case study. Hosted by the Kingdom of Saudi Arabia (KSA), Hajj is one of the largest mass gathering events globally. Muslim pilgrims perform the annual Hajj in the city of Mecca in KSA from 8th to 12th of Dhul-Hijja, the 12th month of the Islamic lunar calendar. Although pilgrims stay together during the 5-day period to follow the rituals, most of them spend three to six weeks in Mecca and Medina before and/or after the event. Medina is another holy place for Muslims, 450km away from Mecca. Over two million pilgrims attend Hajj every year. Their movement within Mecca and to Medina is regulated, yet all pilgrims gather in Mecca for the 5-day ritual. The overcrowding and intense congestion in Hajj has indeed caused outbreaks among pilgrims and the local population. During the 2000 and 2001 Hajj pilgrimages, outbreaks of IMD had occurred and led to global spread of the responsible strain [6, 8].

IMD is caused by *Neisseria meningitidis* leading to severe disease, complication and long term sequelae or even death. *N. meningitidis* carriage and transmission are particularly high in crowded conditions and close contacts [9]. The demographics of pilgrims is also an important factor for disease transmission [8]. Many Hajj pilgrims originate from the meningitis belt, a region in central Africa where major outbreaks of IMD are occurring [10]. A majority of these pilgrims are elderly potentially impacted by immunosenescence [11].

Following the 2000–2001 outbreak, the Hajj Monitoring Authority (HMA) initiated several measures including mandatory vaccination with a quadrivalent vaccine against serogroups A, C, W and Y, prophylactic treatments, masks, counseling, real-time surveillance and sharing of data [12, 13]. These preventive measures were successful to contain many outbreaks. However, the risk of a major transmission or outbreak still remains due to various unpredictable factors e.g. introduction of novel pathogens or new strains, reduction of vaccine coverage for vaccine-preventable diseases, deterioration in the other preventive measures. Indeed, COVID-19 pandemic led KSA to cancel Hajj pilgrimage of 2020 [14, 15].

In this paper, we aim to provide better understanding of the transmission mechanisms during Hajj and insights regarding the effectiveness of vaccination both for local and pilgrim population.

## Methods

The analysis performed is based on a compartmental, meta-population, and age-structured model representing the transmission dynamics of meningococcal infection. This model was specifically designed for assessing the impact of Hajj pilgrimage on disease transmission and calibrated with historical IMD data in Saudi Arabia.

### Mathematical model

The world population, including both pilgrims and non-pilgrims people, is modeled in homogeneous groups called clusters. Each cluster shares the same representation of the infection and demographic processes. The Hajj-specific impact is mainly represented through interactions between the different clusters included in the model. A full mathematical description of the model is given in Additional file 1: Text 1.

Following Hethcote [16], we consider a population with a continuous flow of individuals. In keeping with several previous publications [17, 18], we simplified *N. meningitidis* transmission by aggregating all serogroups. This facilitated a stronger focus on the consequences of MGs. Regarding the infection process, an important specificity relates to the distinction between short-term and asymptomatic carriage. This enables a better representation of the time between infection and disease occurrence which is an important factor for time-limited events such as Hajj pilgrimage.

Hajj-related transmission is first represented by dividing the population of each cluster at the start of each Hajj period between pilgrims and non-pilgrims. The proportion of pilgrims is both cluster and age-dependent. We also account for Hajj vaccination that occurs in the model at the time pilgrims are transferred to the pilgrim-specific states. At the end of a given Hajj period, subjects in pilgrim-specific compartments are reintegrated in the non-pilgrim corresponding compartment. It is worth mentioning that this representation is a simplification compared to the actual pilgrimage process. We notably consider that pilgrims all begin and end their pilgrimage at the same time and did not account for differences in the propensity to become a pilgrim according to disease status. The demographic process is unchanged for pilgrim-specific compartments. Pilgrims can change age group or die during the Hajj. We however restricted births to non-pilgrims compartments. We also account for an increased risk of carriage acquisition for the local population of the Hajj site linked to contacts with pilgrims.

### Data

This study is supported by various data sets including population demography, outbreak cases and incidence, disease prevalence, vaccine schedule, Hajj schedule, annual number of pilgrims and their characteristics (age, immunization, etc.). Population demography and carriage rates are used to calibrate endemic equilibrium and outbreak data are used to calibrate model parameters and validate the model predictions. Detailed information on data used are presented in Additional file 1: Text 1.

The Hajj pilgrims gather from more than 180 countries which include both low and high meningitis endemic regions (Table 1). A majority of pligrims originate from Asian countries (Additional file 1: Text 1, Table S1). In general, there is a low prevalence in Europe and American countries whereas African countries in which the so called ‘meningitis belt’ falls are highly endemic. Since it is impractical to consider each country separately and include their population in a model to study the disease transmission, we split the globe into several clusters. While there is no fine line that may be drawn to create the homogeneous zones, we divide the participant countries (except KSA) into three clusters (or zones) based on the prevalence of meningitis and infection risk labelled high, medium, and low transmission clusters [19]. In addition, Mecca and KSA outside of Mecca are considered as two separate clusters due to their strategic locations (subject to an increased risk during the Hajj of meningitis transmission for the population belonging to these areas). This gives us a total of 5 clusters shown in Table 1.

**Table 1:** Cluster information

Clusters	Country or territories	Carriage rate (%)	Source
Cluster 1: Mecca	Mecca (Hajj city)	4.2 [2.0, 17.8]	Calibrated
Cluster 2: KSA outside Mecca	Kingdom of Saudi Arabia (Hajj country) except Mecca	1.2 [0.5, 3.2]	Calibrated
Cluster 3: High transmission	African meningitis belt countries (Benin, Burkina Faso, Cameroon, Central African, Republic,Chad, Ivory Coast, Congo, Democratic Republic of Congo, Ethiopia, Gambia, Guinea, Ghana, Mali, Mauritania, Niger, Nigeria, Senegal, South Sudan, Sudan, Togo)	6.3	[19]
Cluster 4: Medium transmission	South Africa, Asia (except Turkey, Malaysia, the Philippines, Indonesia, Russia, China), Arabic Non-GCC	4.0	[19]
Cluster 5: Low transmission	Gulf Cooperation Council countries (except KSA), Europe, Americas, Australia, Turkey, Malaysia, The Philippines, Indonesia, Russia, China	2.0	[19]

Carriage rates varies across clusters. For high, medium and low transmission clusters, carriage rates are based on published estimates [19] and derived from calibration results for the two KSA clusters. For the 1995–2001 period, the estimated carriage rate is respectively 4.2% [2.0; 17.8] for Mecca and 1.2% [0.5–3.2] for the rest of KSA. For these two clusters, the range of variation reflects year-to-year variation in meningitis transmission.

### Calibration of transmission parameters

All transmission parameters were calibrated. The first calibration step allowed us to obtain cluster-specific transmission parameters while the second step focused on Hajj-specific transmission parameters.

Calibration of cluster-specific transmission parameters $(\beta _c)$, was based on available evidence on the proportion of carriers for each cluster given its level of endemicity (high, medium or low). We used the analytic solution for the endemic equilibrium in the absence of vaccination at the cluster level to adjust $(\beta _c)$ to these proportions. These endemic equilibria were further used for initializing the model. We considered a burn-in period of 95 years before the period used for calibrating Hajj-related parameters.

Three type of Hajj-related parameters were calibrated: Hajj density effect $\beta _{H}$, impact of Hajj on local transmission $\beta _{L}$ and year-to-year variation in meningitis transmission in KSA clusters $\beta _y$. The calibration of Hajj-related parameters was based on a comparison between model outcomes and historical annual data on IMD in KSA in 3 groups: pilgrims, local population in Mecca and local population in the rest of KSA. The period with available data was divided in 2 periods: a calibration period directly used for fitting parameters values and a validation period used for assessing model accuracy. To get a range of possible values for Hajj-related parameters, we used 100 random samples assuming that each observed data point was Poisson distributed with a mean corresponding to the observed value. Hajj-related parameters were fitted for each of these random samples through the maximization of a quasi log-likelihood function.

### Model simulations

For each scenario considered in the simulations, we generated at least 100 samples using for the transmission parameters the set of possible values obtained through calibration. For each of this scenario we calculated both the median values and 95% credible intervals for the number of IMD cases for specific clusters or specific population (pilgrims and non-pilgrims). We also calculated probability for the number of IMD cases to exceed a given threshold.

## Results

### Calibration results

The results of the model calibration to the IMD cases observed in KSA over the 1995–2001 period presented in Table 2 first highlight the significant increase in disease transmission during the Hajj period. Compared to the non-Hajj period, the density effect associated to Hajj ($\beta _H$) was estimated to generate a 78-fold increase in disease transmission. This major increase reflects the large differences in incidence observed between pilgrims, despite the short Hajj period (20 days as considered here), and the non-pilgrims in KSA for which the period of exposure is the entire year (Additional file 1: Fig. S2). The estimated parameters also indicate significant year-to-year variation in disease transmission in the range of 1–10 ($\beta _y$ ranging from 0.262 to 2.092) that results from the large variation in the number of IMD cases reported in KSA over the 1995–2001 period.

**Table 2:** Hajj-related calibrated parameters

Parameter	Description	Central estimate	Range
$\beta _H$	Hajj density effect	78.5	[68.5, 89.6]
$\beta _L$	Increased risk for the local population	0.125	[0.06, 0.255]
$\beta _y$	Year-to-year transmission variability	0.808	[0.262, 2.092]

**Fig. 1:**
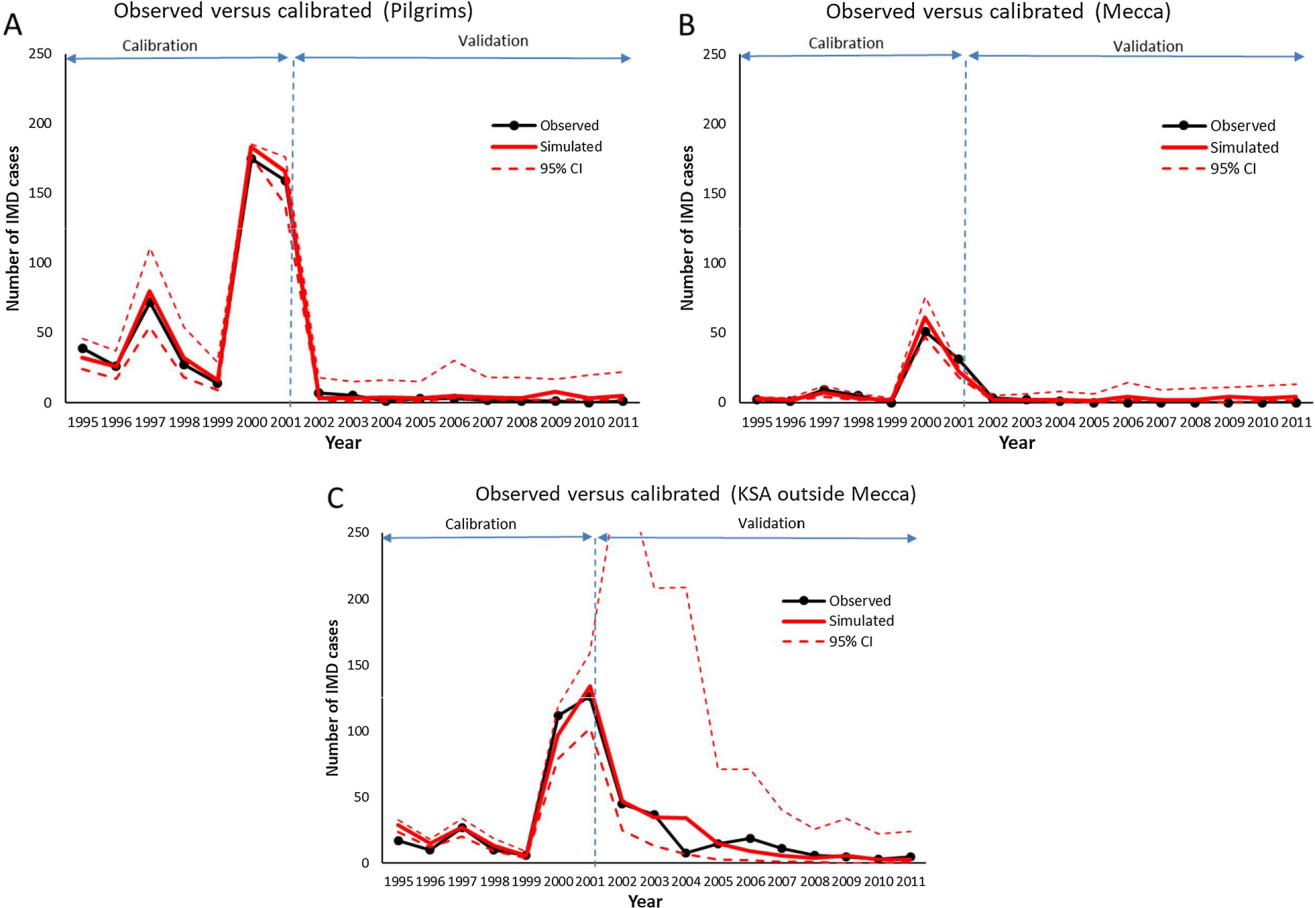
Observed and simulated number of IMD cases (**A**) pilgrims, **B** non-pilgrim population in Mecca (**C**) non-pilgrim population in KSA outside Mecca. Calibration period (1995–2001), validation period (2002–2011)

The calibration procedure enabled to reproduce satisfactorily (Fig. 1) the observed number of IMD cases in the 3 groups considered in the analysis (Pilgrims, Mecca residents and rest of the KSA population) not only for the period used for the estimation but also the period used for validation (2002–2011). In this second period, quadrivalent ACWY vaccines were made mandatory for all the pilgrims and the residents of Mecca and Medina and routine and catch-up vaccination programs were also implemented for the entire population.

### Vaccine impact of low coverage and supply shortage

**Fig. 2:**
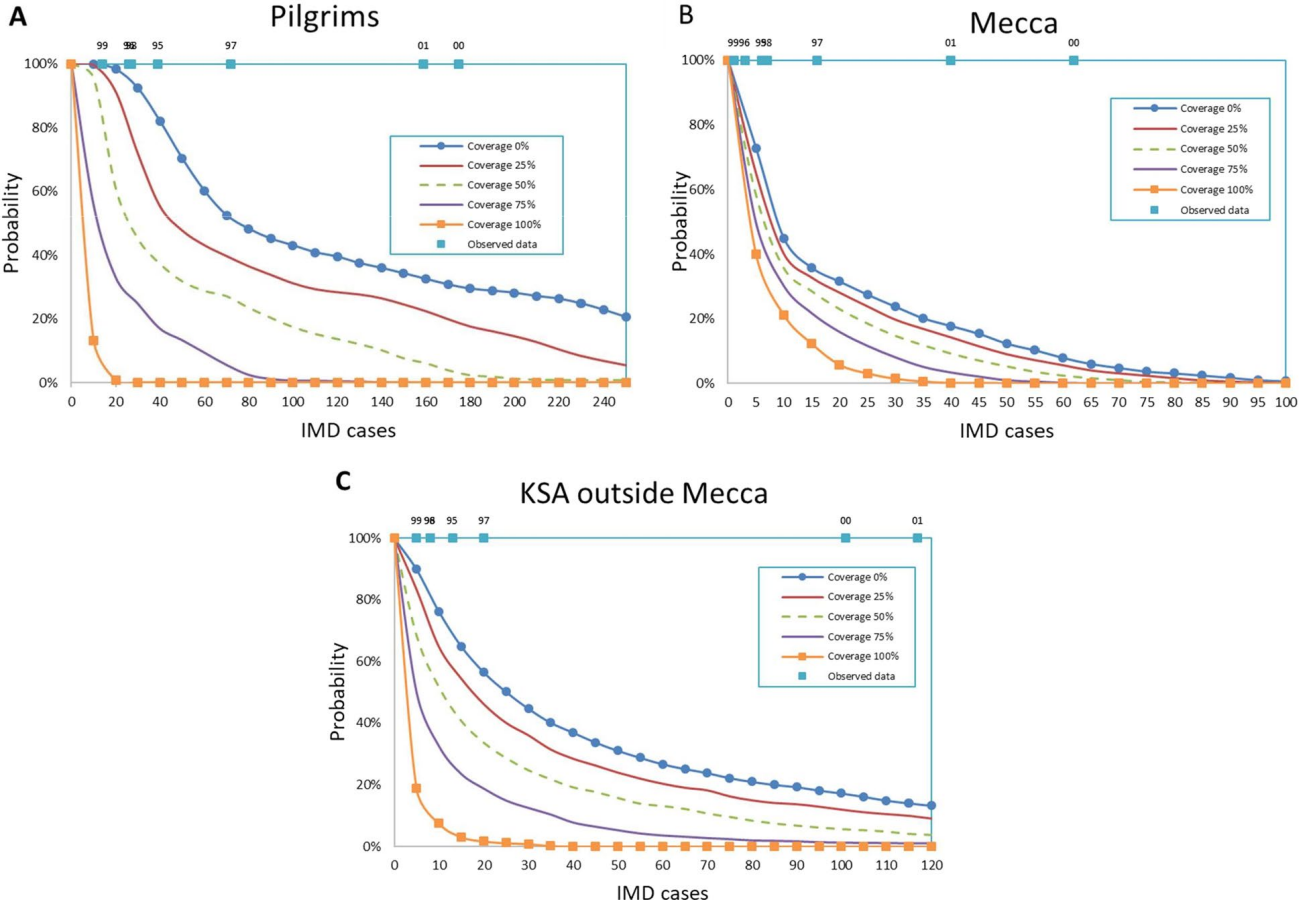
Reverse cumulative distribution of annual IMD cases according to pilgrim vaccination coverage. **A** pilgrims, **B** non-pilgrim population in Mecca (**C**) non-pilgrim population in KSA outside Mecca. Distributions are calculated over a 20 years period

Under the current Hajj guidelines [12], the pilgrims are required to have recommended vaccine. Since 2002, a quadrivalent ACWY meningococcal vaccine is a requirement for all Hajj pilgrims [20]. A lack of compliance to this recommendation might directly impact the number of IMD cases not only among pilgrims but also for the local population of Mecca and the rest of KSA. Figure 2 shows how the decrease of coverage rate could lead to outbreaks, with historical comparatives, through the reverse cumulative distribution curve of annual IMD cases among pilgrims and non-pilgrims for vaccination coverage varying from 0 to 100%. In the absence of vaccination, we estimated a 50% probability of at least 80 IMD cases among pilgrims in a single year (Fig. 2A), at least 10 cases among non pilgrims in Mecca (Fig. 2B) and 20 cases among non pilgrims in the rest of KSA (Fig. 2C). Even with a 75% vaccine coverage, the risk of a meningitis outbreak of more than 30 cases among pilgrims remain significant (25%). On the other side, a full compliance to mandatory pilgrim vaccination make the risk of observing more than 20 IMD cases among pilgrims in a single year very limited ($<1\%$).

**Fig. 3:**
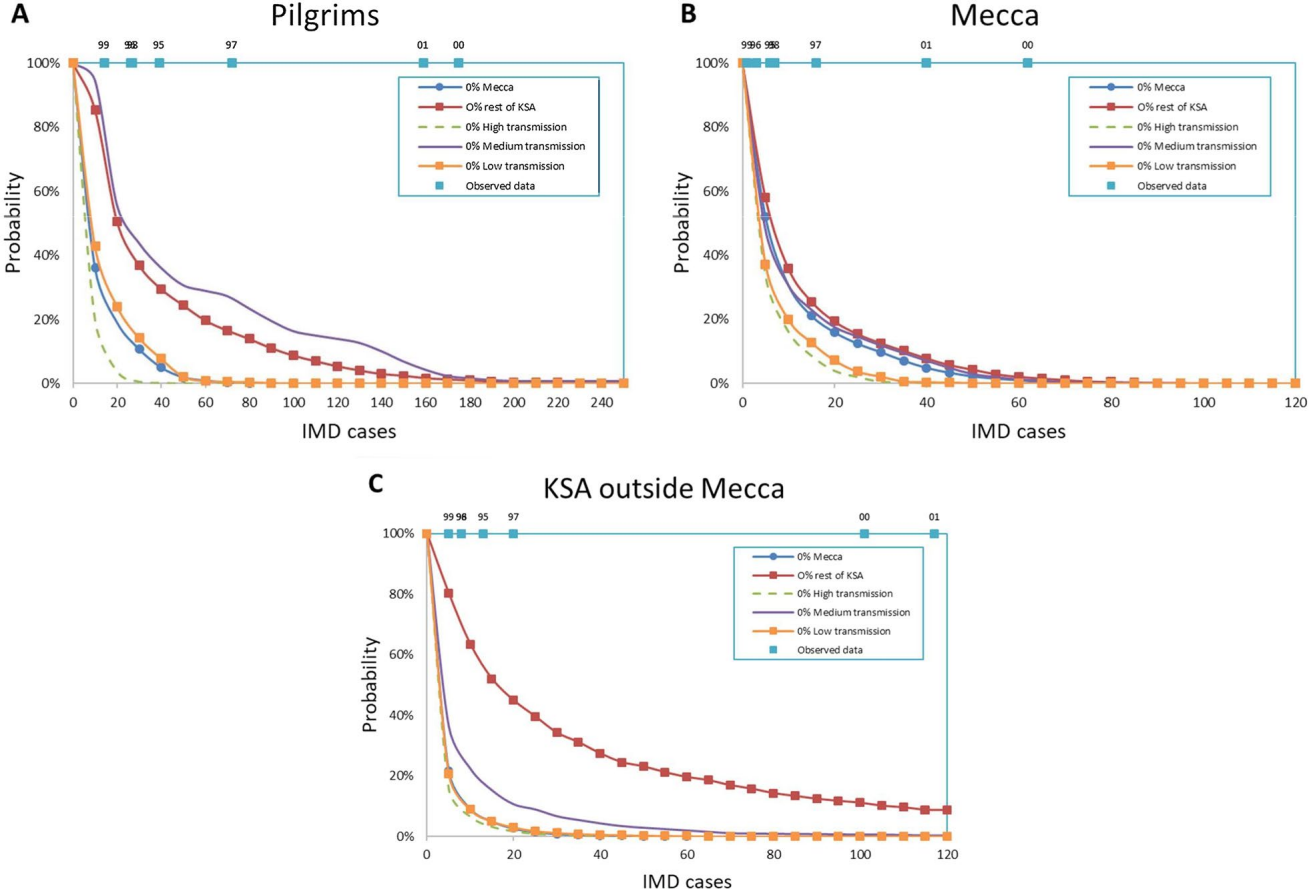
Reverse cumulative distribution of annual IMD cases in case of vaccine shortage in one cluster. **A** pilgrims, **B** non-pilgrim population in Mecca (C) non-pilgrim population in KSA outside Mecca. Distributions are calculated over a 20 years period

Next, we looked into a scenario where pilgrims from one specific cluster are not vaccinated due to supply shortage (Fig. 3). The largest impact was observed when shortage occurs in the medium transmission or KSA outside Mecca clusters, Fig. 3A. This is directly linked to the proportion of pilgrims coming from these two clusters (Additional file 1: Text 1, Table S4). In such scenarios, the non-pilgrims of KSA and Mecca are expected to be only moderately impacted (Fig. 3B,C) except in the shortage occurs in their cluster. The level of pilgrim vaccination coverage also has a limited impact on the number of IMD cases in the clusters outside KSA (Additional file 1: Text 1, Table S8).

### Impact of routine vaccination

We estimated the possible effect of alteration of the current vaccination programs in KSA in place since 2002 on the number of IMD cases in the whole KSA. Table 3 shows the predicted cumulative IMD cases over a 50-year period under three different scenarios; current routine vaccination, routine vaccination in Mecca only and no routine vaccination in KSA. Results show that the IMD cases could be increased by about 40% in the first decade and by more that 50% in the long term. Maintaining routine vaccination only in Mecca is also associated with a significant increase in cases compared to the current situation. The routine vaccination on the opposite has no real impact in the non-KSA clusters (Additional file 1: Text 1, Table S9).

**Table 3:** Impact of routine vaccination on the number of IMD cases per decade in the whole KSA

	Current routine vaccination in KSA (1 year old)	Routine vaccination only in Mecca	No routine vaccination in KSA
2012–2021	99	114	138
	[40, 220]	[41, 301]	[49, 341]
2022–2031	154	233	284
	[62,397]	[77,1113]	[95,1218]
2052–2061 (+40 years)	399	729	853
	[140, 811]	[147, 2331]	[195, 2522]

### Variation of Hajj density effect

**Fig. 4:**
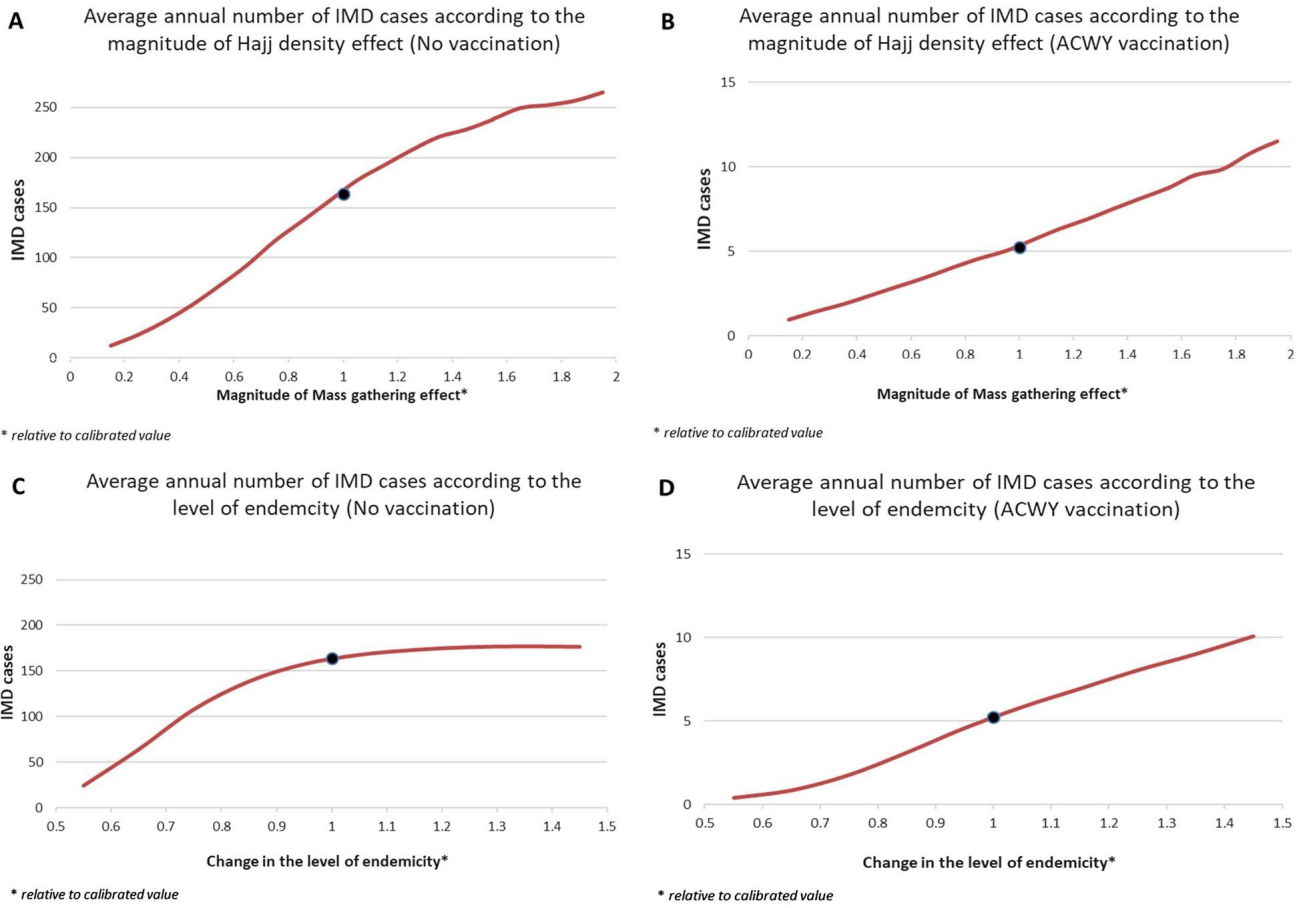
Impact of Hajj density effect and baseline endemicity on IMD cases among pilgrims.Variation of the average annual number of IMD cases among pilgrims (over a 20 years period) (**A**) according to the magnitude of the Hajj density effect in the absence of vaccination (**B**) according to the magnitude of the Hajj density effect with quadrivalent ACWY vaccination for pilgrims and the local population (**C**) according to the level of baseline endemicity in the pilgrim population in the absence of vaccination (**D**) according to the level of baseline endemicity in the pilgrim population with quadrivalent ACWY vaccination for pilgrims and the local population. Black dots corresponding to the number of IMD cases using calibrated values

We explored the impact of a variation of the magnitude of the Hajj density effect on the number of IMD cases among pilgrims using as a reference the level derived from the calibration. Such variation could correspond to potential changes in Hajj likely to impact meningitis transmission and also provides an indirect way to consider other mass gathering events. The impact of this variation is mostly linear notably when pilgrim vaccination is implemented: a density effect 50% lower than the one derived from the calibration translates into a reduction of about 50% of the number of IMD cases among pilgrims with and without vaccination. On the other hand, a 50% increase in the density effect translates into a 56% increase in IMD cases if pilgrim vaccination is implemented but only 30% in the absence of vaccination (Fig. 4).

### Variation of baseline endemicity

Finally, we explored the impact of an change in the level of meningitis carriage impacting all pilgrims. Such variation reflects potential changes in the overall level of meningitis transmission in the countries pilgrims originate from. Such as the Hajj density effect, this variation has mostly a linear impact on the number of IMD cases when pilgrim vaccination is implemented: a 25 % reduction in the proportion of carriers translates into a 70% reduction in the number of IMD cases among pilgrims, whereas a 25% increase translates into a 55% increase (Fig. 4). On the other hand, an increase in endemicity does not lead to a strong increase in the number of IMD cases: a 25 % increase in the proportion of carriers translates into a 5% increase in the number of IMD cases among pilgrims.

## Discussion

Mass gatherings, due to their overcrowded and intense congestion, have the potential to facilitate spread of infectious pathogens. Here, we consider the transmission of *N. meningitidis* during Hajj to develop a general mathematical framework to investigate the impact of vaccines on preventing disease cases, potential outbreaks, and overall transmission during mass gathering events.

Several reasons motivated the selection of this case study. First of all, IMD is a serious disease leading to devastating complications, long term sequelae and death and constitute a major public health issue, particularly when outbreaks occur. Secondly, *N. meningitidis* carriage and transmission are particularly sensitive to crowded conditions and close contacts. Thirdly, not only Hajj is one of the largest mass gathering event worldwide but also IMD in KSA and attendance to Hajj have been monitored for many years and are well documented. We were therefore able to benefit from a total of 17 years of data (1995–2011) for calibrating and validating our model. This period includes occurrence of a large outbreak (2000–2001) and major changes in vaccination policy from 2002.

Our analysis first highlighted the very significant role of Hajj on meningitis transmission. The calibration process identified a 78-fold larger transmission rate during Hajj (Table 2). This magnified transmission rate could be translated to nearly 78-fold new cases compared to the transmission in a regular population with no added density (Fig. 4). The density has a nonlinear effect on the transmission rate and new cases. The cases can be increased by 15–20% if the density increases by 10% from its base value. While this result may not be verified without further data, other studies suggest that mass gathering could magnify an outbreak significantly [21, 22].

Following model validation, we evaluated the impact of the quadrivalent ACWY meningococcal vaccine on the transmission among the pilgrims and non-pilgrims. It suggests that in the absence of vaccination the risk of large outbreak is significant (17% probability to observe more than 100 IMD cases). However, under current vaccine policy (100% coverage) our analysis predict that hardly 10 IMD cases may occur. In addition, we assessed the possibility of vaccine supply shortage and attendance of unvaccinated pilgrims from one cluster. In this scenario, the outbreak seems to be less significant unless the unvaccinated pilgrims are traveling from the medium endemicity cluster which may result in about 25 cases. A possible reason for this could be the number of participants in the Hajj from this cluster that represent about half of all pilgrims.

The results also suggest that pilgrim vaccination may significantly affect the residents of Mecca due to their close proximity with the pilgrims (Fig. 2). Every outbreak in the Hajj has an immediate consequence in Mecca. IMD outbreaks in Hajj would spread to Mecca residents and may not affect the entire region of KSA. These results are consistent with the previous outbreaks [23]. To protect the local residents, a routine quadrivalent ACWY meningococcal vaccination has been implemented in Mecca and KSA since 2002 and the drastic reduction in IMD cases observed the following years provide a direct indication of the important role of vaccination. Our simulation indicates that routine vaccine in the hosting country is crucial to prevent any local outbreaks. Stopping the routine vaccination program may not have a severe impact immediately, but in its absence, the IMD cases may rise gradually and the number can be quadrupled in 40 years, Table 3. Overall, these results highlight the importance of continuing the current vaccination policy for the pilgrims and local residents. However, the risk of a major transmission or outbreak still remains due to various unpredictable factors including the introduction of novel pathogens or new strains, and reduction of vaccine coverage.

Our study has several limitations. First, we simplified *N. meningitidis* transmission by aggregating all serogroups to focus more specifically on the consequences for Hajj. Our representation of Hajj also does not capture fully the complexity of transmission mechanisms at play : only 5 clusters where considered in our model whereas pilgrims originate from more than 100 countries, we used a standard duration for Hajj pilgrimage (20 days) whereas this duration vary significantly among pilgrims, pilgrims also tends to have more close contact with other pilgrims with the same country of origin. The vaccine characteristics considered in our analysis correspond to a conjugate vaccine whereas either polysaccharide or conjugate vaccines can be used for Hajj pilgrimage [24]. Polysaccharide vaccines are associated with a shorter duration of protection and have no significant impact on carriage [25]. They might therefore have a more limiting ability to prevent outbreaks. However, to meet vaccine requirements, polysaccharide vaccine needs to be administered less than 3 years before Hajj and there is also evidence that such vaccines can reduce transmission [26, 27]. Data used also derive from a variety of sources which raises some consistency and quality issues. Information on meningitis prevalence for instance remains limited. We used a limited period of time for model calibration (7 years) and did not account for vaccination effect during this period whereas some pilgrims had received at that time bivalent vaccination.

## Conclusion

Our analysis, that provides quantitative estimates informed by historical data, highlighted the amplifying effect of mass gathering on disease transmission and confirm vaccination as a very effective preventive measure. Although the results presented here are only applicable to Hajj and *N. meningitidis*, the insights provided might also help informing public health interventions for disease prevention and control for other events and other transmissible diseases, such as COVID-19 that has now become a major concern for mass gathering events.

## Supplementary Information

**Additional file 1:** Text 1 includes a detailed model description, Supplementary data, Supplementary results, FiguresS1, S2, and Tables S1-S9.

## Data Availability

All datasets analysed in this manuscript, based on publicly available information, are presented in detail in supplementary material and are available from the corresponding author upon request.

## Abbreviations

MGs: Mass gathering events
IMD: Invasive meningococcal disease
*N. meningitis*: *Neisseria meningitis*
