# Supplementary material for: Disease transmission and mass gatherings: a case study on meningococcal infection during Hajj
Source: BMC Infect Dis. 2022 Mar 22;22:275. doi: 10.1186/s12879-022-07234-4 (PMC8938638; doi:10.1186/s12879-022-07234-4)
Supplement: Supplementary file 1 — Additional file 1: Text 1 includes a detailed model description, Supplementary data, Supplementary results, FiguresS1, S2, and Tables S1-S9. [file 12879_2022_7234_MOESM1_ESM.pdf]

Disease transmission and mass gatherings: a case study on meningococcal infection during Hajj
Supplementary Text 1

Laurent Coudeville, Amine Amiche, Ashrafur Rahman, Julien Arino, Biao Tang, Ombeline Jollivet, Alp Dogu, Edward Thommes, Jianhong Wu

---

S1. Model description

We developed a compartmental age-structured meta-population model. Below, we present first the demographic and infection processes at the cluster level before detailing the characteristics and interactions between clusters. The flow diagram of the infection and vaccination processes are presented in Figure S1.

S1.1. Demographic process

Following Hethcote[1], we consider a population with a continuous flow of individuals between age groups. Under this representation, the demographic and infection processes can be separated and for individuals in disease status X, age group i and cluster c, one can write

$$\dot{X}_{c,i} = D^X_{c,i} + I^X_{c,i} \tag{S1.1}$$

where $D^X_{c,i}$ and $I^X_{c,i}$ correspond respectively to the demographic process and infection process for compartment X i.e. transition rates between compartments at a given time t due to birth, death or ageing (demographic) or change in disease status (infection).

In the absence of vaccination, the demographic process is identical whatever the disease status and is given for every age group except the first one by

$$D^X_{c,i} = \Theta_{i-1}X_{c,i-1} - (\mu + \Theta_i)X_{c,i} \qquad (i > 1) \tag{S1.2}$$

Θ_i is the transition rate from age group i to age group i+1, defined as follows:

$$\Theta_i = \frac{\mu_i + g}{e^{(\mu_i+g)l_i} - 1}, \tag{S1.3}$$

where g stands for the growth rate of the population, μ the death rate and l_i for the length of age class i. In the first age group, except for the compartment corresponding to disease status at birth, the demographic process simplifies to

$$D^X_{c,1} = -(\mu_i + \Theta_i)X_{c,i}. \tag{S1.4}$$

Figure S1: **Flow diagram of the infection process**

.

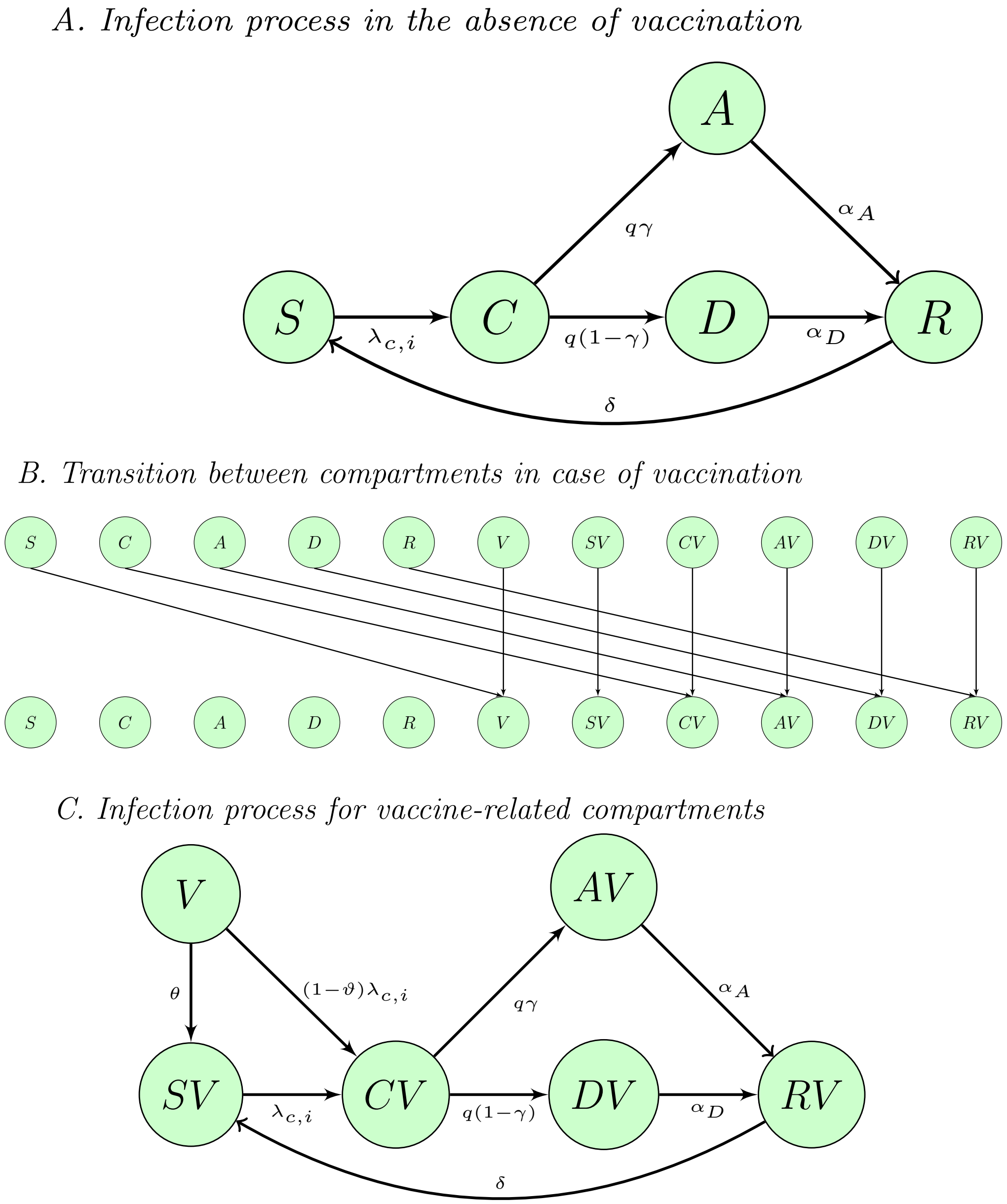

A. Flow diagram in the absence of vaccination, B. Transition between compartments in case of vaccination and C. Flow diagram for vaccine-related compartments. Model compartments : Susceptible(S), Short-term carrier(C), Asymptomatic carrier(A), Diseased(D), Recovered(R), Vaccinated and protected against infection (V), Vaccinated but susceptible to meningococcal infection (SV), Vaccinated and short-term carrier (CV), Vaccinated and asymptomatic carrier(AV), Vaccinated and Diseased (DV), and Vaccinated and immune following infection (RV)

We consider that, in the absence of vaccination, all newborns are susceptible (corresponding to compartment $S_{c,1}$). Therefore

$$D_{c,1}^{S} = B_c - (\mu_i + \Theta_i)X_{c,i}, \tag{S1.5}$$

where B_c is the number of births at date t defined as a fraction of the population size:

$$B_c = f_c \sum_{i=1}^{n_c} P_{c,i}, \tag{S1.6}$$

where $P_{c,i}$ is the population size in age group i and f_c the birth rate in cluster c. At demographic equilibrium, the following equations hold

$$\forall i > 1 \; P_{c,i} = \frac{\Theta_{i-1}P_{i-1}}{\Theta_i + \mu_i + q}; P_{c,1} = \frac{B_c}{\Theta_i + \mu_i + g}; f_c = \frac{P_1(\Theta_1 + \mu_1 + g)}{\sum_{i=1}^{n_c} P_{c,i}}. \tag{S1.7}$$

S1.2. Infection process in the absence of vaccination

In the absence of vaccination, the population in a given age group and cluster can be divided in 5 compartments : Susceptible(S), Short-term carrier(C), Asymptomatic carrier(A), Diseased(D), and Recovered(R).The corresponding set of equations is given by :

$$\begin{cases} I_{c,i}^{S} &= \delta R_{c,i} - \lambda_{c,i}S_{c,i}, \\ I_{c,i}^{C} &= \lambda_{c,i}S_{c,i} - qC_{c,i}, \\ I_{c,i}^{A} &= q\gamma C_{c,i} - \alpha_A A_{c,i}, \\ I_{c,i}^{D} &= q(1-\gamma)C_{c,i} - \alpha_D D_{c,i}, \\ I_{c,i}^{R} &= \alpha_A A_{c,i} + \alpha_D D_{c,i} - \delta R_{c,i}. \end{cases} \tag{S1.8}$$

The force of infection in the absence of vaccination and if transmission only occurs locally in cluster c is given by

$$\lambda_{c,i} = \frac{\sum_{j=1}^{n_c} \beta_{ij}(C_{c,j} + A_{c,j} + \epsilon D_{c,j})}{\sum_{j=1}^{n_c} P_{c,j}}, \tag{S1.9}$$

where $P_{c,j} = S_{c,j} + C_{c,j} + A_{c,j} + D_{c,j} + R_{c,j}$ is the total population in age group j.

By normalizing the force of infection with the size of the population, we considered here a density-dependent transmission [2]. Infectivity is considered to be identical for short-term and asymptomatic carriage but lower for diseased individuals ($\epsilon < 1$). This formulation of the force of infection enables considering age-specific transmission rate (corresponding to the matrix of β_{ij}). However, for sake of simplicity, we assume throughout the analysis presented here homogeneous mixing ($\forall i, j \; \beta_{ij} = \beta_{ji} = \beta_c$).

S1.3. Model with vaccination

If one excepts Hajj-related vaccination, vaccination occurs when people move from one age group to another. This impact the ageing process, since in case of vaccination, the compartment before vaccination ($X_{c,i-1}$) differs from the compartment after vaccination ($X_{c,i}$).

Vaccination is also accounted for in the model through the addition of 6 vaccine-related compartments: Vaccinated and protected against infection (V), Vaccinated but susceptible to meningococcal infection (SV), Vaccinated and short-term carrier (CV), Vaccinated and asymptomatic carrier(AV), Vaccinated and Diseased (DV), and Vaccinated and immune following infection (RV). The infection process is given by

$$\begin{cases} I_{c,i}^{S} &= \delta R_{c,i} - \lambda_{c,i} S_{c,i}, \\ I_{c,i}^{C} &= \lambda_{c,i} S_{c,i} - q C_{c,i}, \\ I_{c,i}^{A} &= q\gamma C_{c,i} - \alpha_A A_{c,i}, \\ I_{c,i}^{D} &= q(1-\gamma) C_{c,i} - \alpha_D D_{c,i}, \\ I_{c,i}^{R} &= \alpha_A A_{c,i} + \alpha_D D_{c,i} - \delta R_{c,i}, \\ I_{c,i}^{V} &= -\theta V_{c,i} - (1-\vartheta)\lambda_{c,i} V_{c,i}, \\ I_{c,i}^{SV} &= \theta V_{c,i} + \delta RV_{c,i} - \lambda_{c,i} SV_{c,i}, \\ I_{c,i}^{CV} &= \lambda_{c,i} SV_{c,i} + (1-\vartheta)\lambda_{c,i} V_{c,i} - qCV_{c,i}, \\ I_{c,i}^{AV} &= q\gamma CV_{c,i} - \alpha_A AV_{c,i}, \\ I_{c,i}^{DV} &= q(1-\gamma) CV_{c,i} - \alpha_D DV_{c,i}, \\ I_{c,i}^{RV} &= \alpha_A AV_{c,i} + \alpha_D DV_{c,i} - \delta RV_{c,i}. \end{cases} \tag{S1.10}$$

The force of infection becomes:

$$\lambda_{c,i} = \frac{\sum_{j=1}^{n_c} \beta_c (C_{c,j} + A_{c,j} + CV_{c,j} + AV_{c,j} + \epsilon D_{c,j} + \epsilon DV_{c,j})}{\sum_{j=1}^{n_c} P_{c,j}}. \tag{S1.11}$$

S1.4. Hajj-related transmission

Hajj-related transmission is first represented by dividing the population of each cluster at the start of each Hajj period between pilgrims and non-pilgrims. The proportion of pilgrims is both cluster and age-dependent. For instance, the proportion of individuals in disease status X in cluster c and age group i transferred at the start of a given Hajj period ($t = t_{hs}$) to the pilgrim-specific compartments associated to cluster c is given by:

$$\begin{cases} X_{c,i}^{p} &= h_{c,i} X_{c,i} \\ X_{c,i} &= (1-h_{c,i}) X_{c,i} \end{cases} \tag{S1.12}$$

We also account for Hajj vaccination that occurs in the model at the time pilgrims are transferred to the pilgrim-specific states. The vaccination process is otherwise similar to the one described previously. At the end of a given Hajj period ($t = t_{he}$), subjects in pilgrim-specific compartments are reintegrated in the non-pilgrim equivalent compartment:

$$\begin{cases} X_{c,i}^{p} &= 0 \\ X_{c,i} &= X_{c,i} + X_{c,i}^{p} \end{cases} \tag{S1.13}$$

The demographic process is unchanged for pilgrim-specific compartments. Pilgrims can change age group or die during the Hajj. We however restricted births to non-pilgrims compartments.

The infection process for pilgrims is also identical to the one defined in S1.10. The only difference relates to the increased contact rates between pilgrims (Hajj density effect) that led to the specific force of infection for pilgrims during Hajj ($t_{hs} < t < t_{he}$):

$$\lambda_p = \frac{\sum_{c=1}^{N} \sum_{j=1}^{n_c} \beta_H \beta_y \beta_c (C_{c,j}^p + A_{c,j}^p + CV_{c,j}^p + AV_{c,j}^p + \epsilon D_{c,j}^p + \epsilon DV_{c,j}^p)}{\sum_{c=1}^{N} \sum_{j=1}^{n_c} P_{c,j}^p} \quad \text{(S1.14)}$$

β_H corresponds here to the specific Hajj density effect defined as a multiplicative factor. In addition, we also consider year-to-year variation in the level of meningitis transmission which corresponds to β_y in (S1.14)

We also account for an increased risk of carriage acquisition for the local population of the Hajj site linked to contacts with pilgrims (β_L). If we consider that the first cluster considered in the model corresponds to the population of the Hajj site (c=1). The force of infection during Hajj for the local population is given by

$$\lambda_1 = \frac{\sum_{j=1}^{n_1} \beta_y \beta_1 (C_{1,j} + A_{1,j} + CV_{1,j} + AV_{1,j} + \epsilon D_{1,j} + \epsilon DV_{1,j})}{\sum_{j=1}^{n_1} P_{1,j}} + \beta_L * \lambda_p \quad \text{(S1.15)}$$

The model was coded in C# and the results were generated and analyzed through a web-based simulation platform called DENMOD.

S2. Supplementary data

S2.1. Demography

Population is derived from WHO 2017 population assessment [3] for clusters outside KSA (high, medium and low transmission). We estimated the average annual growth rate per cluster, based on 2000-2019 demographics evolution per country [4]. Demographic data for Mecca and KSA area are based on official KSA statistics[5].

For Mecca and KSA outside Mecca, the whole populations are modeled. For the three other clusters, we only model one tenth of their real population. As the number of Hajj pilgrims compared to the size of these three clusters' population is very small, this choice does not impact the dynamic of the meningitis's spread due to the Hajj and the relative proportions between the three clusters are respected.

Age groups are the same for all clusters. There are twelve age groups: an age group from birth to one year old, an age group from 1 to 5 years old, then 5 age groups of 5 years until 30 years old, then 3 age groups of 10 years until 60 years old and finally 2 age groups of 20 years covering people until 100 years old. Ageing of the population is based on life tables provided by WHO [6]. They are specific to a

country, so the larges country in the cluster was selected to represent the ageing of the population, cf Table S3 i.e. the one with largest impact on the representativeness of the cluster.

Table S1: **Typical Hajj participants from different countries or regions, extracted from 2017 Hajj**

Countries or regions	No. of pilgrims
Africa except RSA	183373
RSA	3500
Arab Non-GCC	383044
Asian Non-Arab	653298
GCC	32600
KSA	600103
Europe	84894
Americas & Australia	22267
Turkey	84637
Malaysia	41200
The Philippines	6000
Indonesia	221000
Russia	23500
China	12700

Source :KSA General Authority for Statistics[7]. Asian Non-Arab corresponds to Asian countries except Turkey, Malaysia, the Philippines, Indonesia, Russia, and China

Table S2: **Annual population of the five clusters during the calibration and validation periods (1995-2011)**

Years	Mecca	KSA (outside Mecca)	High transmission	Medium transmission	Low transmission
1995	315607	397484	52315	784730	209261
1996	347331	437438	54023	810349	216093
1997	342680	431580	58430	876443	233718
1998	309711	390059	56617	849258	226469
1999	338700	426568	52837	792548	211346
2000	201569	253861	63368	950516	253471
2001	195097	245711	68200	1022994	272798
2002	261383	329193	67709	1015638	270837
2003	218299	274931	71551	1073259	286202
2004	209347	263657	70985	1064780	283941
2005	453757	571474	76738	1151077	306954
2006	210756	265431	82720	1240805	330881
2007	330398	416113	85391	1280861	341563
2008	392474	494292	86492	1297381	345968
2009	401872	506128	80650	1209750	322600
2010	466819	587925	89980	1349701	359920
2011	486637	612885	91410	1371146	365639

Source : Mecca and KSA outside Mecca [5] ; Low, medium an high transmission clusters [3, 4]

Table S3: **Demography of the five clusters**

	Mecca	KSA (outside Mecca)	High transmission	Medium transmission	Low transmission
Population (1995)	1005501	17154394	32707426	220500722	316607677
Growth rate(%)	2.70	2.60	2.72	1.21	0.82
Age group(%)					
0-20 years	37.14	45.77	41.21	37.14	39.03
21-30 years	12.15	13.86	12.78	12.15	12.45
31-40 years	11.96	11.77	11.20	11.96	12.01
41^+ years	38.74	28.59	33.80	38.74	36.50
Life table	KSA	KSA	Sudan	India	China
Pilgrims(%)	25.2	1.9	0.17	0.43	0.08

Source : Population size[3], Growth rate[4], age distribution[6]

S2.2. Pilgrimage

Pilgrimage is modeled as a twenty days event, occurring every 354 days. 20 days cover both the period of the religious events - 5 days - and the progressive arrival and departure of pilgrims. Real start dates of the pilgrimage were taken as initial dates of the 20-days period for calibration and validation years (1995-2012) and are automatically computed in simulations. As the time between two Hajj is shorter than a Gregorian year, it occurs that 2 Hajj happen the same Gregorian calendar year, like in 2006. For easiness of reading, all results are based on Gregorian calendar years.

During pilgrimage, the part of the population of each cluster that represent the pilgrims is modeled as a dedicated cluster. The transition mechanism of pilgrims from local population to their dedicated cluster, mimicking the travel from their residency country to the Hajj site, is described in equations (S1.12) and (S1.13). The annual number of pilgrims is taken from KSA Hajj statistics [7] and is used to calculate the proportion ($h_{c,i}$) of pilgrims. Obviously, the fractions of the pilgrims in the clusters are small as shown in Table S3, especially in non-KSA ones. Usually, two to three millions of pilgrims attend the Hajj each year. The typical number of pilgrims and their country of origin can be seen in Table S4. Age distribution of the pilgrims differs from the age distribution of non-pilgrims but remains stable over time[8] and is described in Table S4.

Table S4: **Annual number of pilgrims from the five clusters and their age distribution**

Years	Mecca	KSA (outside Mecca)	High transmission	Medium transmission	Low transmission
1995	315 606.65	397 483.62	52 315.35	784 730.25	209 261.40
1996	347 330.94	437 438.06	54 023.25	810 348.75	216 093.00
1997	342 679.76	431 580.24	58 429.55	876 443.25	233 718.20
1998	309 711.23	390 058.77	56 617.20	849 258.00	226 468.80
1999	338 699.99	426 568.01	52 836.50	792 547.50	211 346.00
2000	201 568.78	253 861.22	63 367.75	950 516.25	253 471.00
2001	195 097.23	245 710.77	68 199.60	1 022 994.00	272 798.40
2002	261 383.06	329 192.94	67 709.20	1 015 638.00	270 836.80
2003	218 298.69	274 931.31	71 550.60	1 073 259.00	286 202.40
2004	209 346.86	263 657.14	70 985.30	1 064 779.50	283 941.20
2005	453 757.03	571 473.97	76 738.45	1 151 076.75	306 953.80
2006	210 755.62	265 431.38	82 720.35	1 240 805.25	330 881.40
2007	330 398.33	416 112.67	85 390.70	1 280 860.50	341 562.80
2008	392 473.70	494 292.08	86 492.05	1 297 380.75	345 968.20
2009	401 871.76	506 128.24	80 650.00	1 209 750.00	322 600.00
2010	466 819.19	587 924.81	89 980.05	1 349 700.75	359 920.20
2011	486 637.49	612 884.51	91 409.75	1 371 146.25	365 639.00
Age group(%)					
20-30 years	8.33	8.33	8.33	9.09	9.09
31-40 years	16.67	16.67	16.67	9.09	9.09
41^+ years	75	75	75	81.82	81.82

Source : Annual number of pilgrims[7], Age distribution[8, 9]

S2.3. Disease

When infected, an individual may progress rapidly to the disease state with symptoms within a week or may carry the infection in an asymptomatic state for several months [10]. From the 2000-2001 Hajj outbreak we noticed a very short period of carriage [11, 12]. The IMD cases were reported in a week or two following the Hajj. From this study we consider the short term carriage to be a week (i.e. $q = 1$). We estimated the long-term (asymptomatic) carriage to 1 year, taking an average value from the 9 to 15 months period evoked in Bennet et al. review[13], consistent with 11.1 up to 14.5 months of carriage estimated by De Wals et al. in [14]. The infectious period of IMD cases is about a week which yields $\alpha_D = 52$ [10].

The waning rate of recovery-induced-immunity was set to 12 years [15], which yields $\delta = 0.0839$/year. No reliable data are found for the relative transmission scaling (ϵ). We assume $\epsilon = 0.5$. All these parameters are summarized in Table S5.

Table S5: **Disease and vaccine parameters**

Parameters	Definitions	Values	Reference
q	Rate of moving out from short-term carriage status (year^{-1})	52	[10]
γ	Proportion of short-term carriers remaining asymptomatic (%)	99.98	[13]
α_A	Recovery rate from asymptomatic carrier status (year^{-1})	1	[14, 13]
α_D	Recovery rate from disease status (year^{-1})	52	[10]
ϵ	Relative infectiousness IMD modification parameter	0.5	Assumed
δ	Waning rate of recover-induced immunity (year^{-1})	0.0839	[**?**]
ϑ	Vaccine efficacy (%)	93	[16]
θ	Waning of vaccine induced immunity (year^{-1})	0.1	[15, 17]
	Age for routine vaccination schedule in KSA (year)	1	[18]
	Routine vaccination coverage rate (%)	96	[19, 20]

S2.4. Vaccine

In 2002, upon available, the polysaccharide quadrivalent ACWY vaccines were made mandatory for all the pilgrims and the residents of Mecca and Medina who are older than two years [21]. KSA also introduced routine vaccines for the toddlers at the age of 9 months[18], still accurate. We model that as routine vaccine for the Mecca and KSA clusters at the age of one year for all simulated years. One is a proxy for 9 months due to age structure of the model and the fact it is the moment where vaccination scheme ends and is efficient. Coverage rate was set to 96%, estimated from analysis of other diseases coverage rate, always very high in KSA[19, 20]. For 2002-2011 (validation period), we take into account children and adult vaccination decided by KSA as a massive vaccination of 90% of Mecca population. From 2012, and all the predictive simulated years, we consider the current situation, so only the vaccinations of babies as detailed above.

Pilgrims from all the five clusters are considered vaccinated according to the Hajj guidelines and regulation [22]. Since there is no consistent and routine vaccines throughout the non-KSA clusters, and that all pilgrims are vaccinated and we study here the impact mainly on pilgrims and on mass gathering event local site, we do not include routine vaccines in those clusters.

Our model is calibrated over the 1995-2001 period. We do not consider any vaccination during this period as vaccination program for pilgrims was only bivalent A/C, that did not cover all circulating strains as outbreaks in 2000 and 2001 demonstrate.

Model is calibrated on the number of cases both in pilgrims and in Mecca and KSA local clusters.

The quadrivalent vaccine is used worldwide from 2002 for the local population and pilgrims against the meningococcal infection which is found to be 93% effective [16]. This allows us to estimate the vaccine efficacy ϑ to be 0.93. Other studies considered $0.6 - 0.9$ for ϑ [15]. Following Karachaliou et al.[15], the waning rate of vaccine-induced-immunity was set to $\theta = 0.1$/year.

S2.5. Hajj outbreaks, IMD cases, and annual incidence

During the past decades several large meningitis outbreaks occurred in the Hajj along with some sporadic cases. Yezli, et al. reported an annual IMD cases in Mecca and KSA for the period of 1987-2015 [23]. They did not distinguish the cases in terms of pilgrims and non-pilgrims. Using the proportion of Hajj participants and estimation reported by Memish et al.[21], we approximate the pilgrims and non-pilgrims cases. Based on these studies, we also estimate the annual number of cases (pilgrims and non-pilgrims) in KSA and Mecca clusters for the calibration period as shown in Table S6. From these observed annual IMD cases we estimate the annual incidence rates (number of cases per 100000 population) for the pilgrims and residents of KSA and Mecca clusters over the period of 1995-2011, shown in Figure S2.

Table S6: **Annual cases in Mecca and KSA**

Year	Pilgrims (all clusters)	Mecca	KSA (outside Mecca)
1995	39	2	17
1996	26	1	10
1997	72	9	27
1998	27	5	10
1999	14	0	6
2000	175	51	112
2001	159	31	126
2002	7	3	45
2003	5	2	37
2004	1	1	8
2005	3	0	15
2006	3	0	19
2007	2	0	11
2008	1	0	6
2009	1	0	5
2010	0	0	3
2011	1	0	5

Source : Derived from data reported in Memish et al.[21] and Yezli et al. [23]

Figure S2: **Annual IMD incidence in Hajj pilgrims, KSA and Mecca regions over the period of 1995-2011**

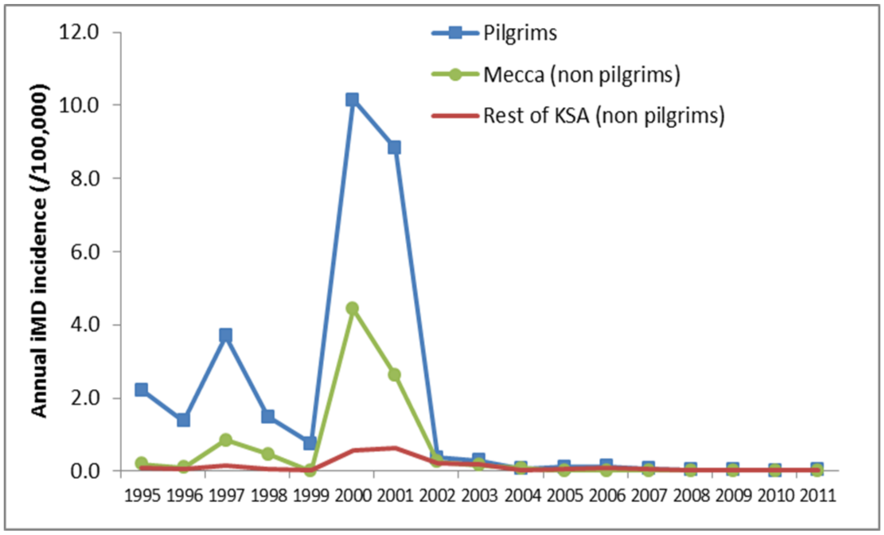

Source : Derived from data reported in Memish et al. [21] and Yezli et al. [23]. Note that the incidence is calculated on annual basis and do not account for differences in actual duration of exposure between pilgrims (Hajj period) and local population (year-round)

S3. Supplementary results

Table S7: **Number of IMD cases among pilgrims over 20 years in each cluster according to the level of pilgrim vaccination coverage**

	Mecca	KSA (outside Mecca)	High transmission	Medium transmission	Low transmission
Base case (100% coverage)	21 [13, 32]	34 [20, 55]	21 [14, 32]	22 [14, 34]	27 [16, 42]
50% coverage in Medium cluster	23 [14, 37]	38 [22, 63]	23 [15, 37]	80 [49, 121]	30 [17, 48]
50% coverage in all clusters	44[31, 64]	298[204, 451]	124[86, 181]	149[102, 214]	193[131, 278]
No vaccine	189[146, 222]	758[510, 977]	466[300, 624]	584[378, 791]	767[500, 1038]

Top row: 100% coverage across all the clusters. Second row: 50% coverage in the Medium cluster and 100% coverage in the other clusters. Third row: 50% coverage in all the clusters. Botton row: No vaccination in all clusters.

Table S8: **Number of IMD cases among non-pilgrims over 20 years in each cluster according to the level of pilgrim vaccination coverage**

	Mecca	KSA (outside Mecca)	High transmission	Medium transmission	Low transmission
Base case (100% coverage)	129 [63, 255]	53 [15, 157]	17614 [17608, 17624]	49667 [49655, 49691]	30983 [30936, 31069]
50% coverage in Medium cluster	153 [77, 297]	67 [17, 215]	17616 [17608, 17631]	50217 [50151, 50324]	31014 [30942, 31145]
50% coverage in all clusters	272 [155, 455]	467 [137, 1146]	17947 [17893, 18017]	50294 [50175, 50466]	32605 [32040, 33284]

Top row: 100% coverage across all the clusters. Middle row: 50% coverage in the Medium cluster and 100% coverage in the other clusters. Bottom row: 50% coverage in all the clusters.

Table S9: **Number of IMD cases in each cluster over the 50 years according to the routine vaccination program in KSA**

	Mecca	KSA (outside Mecca)	High transmission	Medium transmission	Low transmission
Base case	0.7 K [0.5,1]	0.5 K [0.3, 5.8]	71 K [71, 71]	151 K [151,151]	89 K [89,89]
Mecca only	0.7 K [0.4,1]	1.3 K [0.3, 5.8]	71 K [71, 71]	151 K [151,151]	89 K [89,89]
No routine	1.0 K [0.7,1.4]	1.3 K [0.3, 5.8]	71 K [71, 71]	151 K [151,151]	89 K [89,89]

K : Thousands of cases. Top row: IMD cases among the pilgrims in each cluster under a routine vaccination in whole KSA. Second row: IMD cases of pilgrims in each cluster with routine vaccines only in Mecca. Third row: IMD cases with no routine vaccination in KSA.

References

[1] Hethcote H. W. The mathematics of infectious diseases. *SIAM Review*, 42(4):599–653, 2000.

[2] Hu H, Nigmatulina K, and Eckhoff P. The scaling of contact rates with population density for the infectious disease models. *Mathematical Biosciences*, 244:125–134, August 2013.

[3] United Nations . World population prospects: The 2017 revision. `https://population.un.org/wpp/Download/Standard/Population/`, 2017. Accessed: 2019-09-18.

[4] United Nations Population Fund . World population dashboard. `https://www.unfpa.org/data/world-population-dashboard`, 2019. Accessed: 2019-09-18.

[5] KSA General Authority for Statistics . Demographic statistics. `https://https://www.stats.gov.sa`, 2020. Accessed: 2020-07-20.

[6] Global Health Observatory data repository, World Health Organization . Life tables by country. `http://apps.who.int/gho/data/node.main.LIFECOUNTRY?lang=en`, 2018. Accessed: 2019-05.

[7] KSA General Authority for Statistics . Hajj statistics. `https://https://www.stats.gov.sa/en/893-0`, 2017. Accessed: 2020-07-09.

[8] Ebrahim S. H, Memish Z. A, Uyeki T. M, Khoja T. A, Marano N, and McNabb S. J. Public health. Pandemic H1N1 and the 2009 Hajj. *Science*, 326(5955):938–940, Nov 2009.

[9] Lessler J, Rodriguez-Barraquer I, Cummings D. A. T, et al. Estimating potential incidence of MERS-CoV associated with Hajj pilgrims to Saudi Arabia, 2014. *PLoS currents*, 6, November 2014.

[10] Crum-Cianflone N and Sullivan E. Meningococcal vaccinations. *Infectious Diseases and Therapy*, 5:89–112, June 2016.

[11] Aguilera J. F, Perrocheau A, Meffre C, and Hahné S. Outbreak of serogroup W135 meningococcal disease after the Hajj pilgrimage, Europe, 2000. *Emerging Infect. Dis.*, 8(8):761–767, Aug 2002.

[12] Lingappa J. R, Al-Rabeah A. M, Hajjeh R, et al. Serogroup W-135 meningococcal disease during the Hajj, 2000. *Emerging infectious diseases*, 9:665–671, June 2003.

[13] Bennett J, Dolin R, and Blaser M. *Mandell, Douglas, and Bennett's Principles and Practice of Infectious Diseases*, volume 1. Elsevier Health Sciences, 2014.

[14] De Wals P and Bouckaert A. Methods for estimating the duration of bacterial carriage. *International journal of epidemiology*, 14:628–634, December 1985.

[15] Karachaliou A, Conlan A, Preziosi M.-P, and Trotter C. Modeling long-term vaccination strategies with MenAfriVac in the African Meningitis Belt. *Clinical infectious diseases : an official publication of the Infectious Diseases Society of America*, 61 Suppl 5:S594–S600, November 2015.

[16] WHO . Meningococcal vaccines: Who position paper, november 2011. *Weekly epidemiological record*, 86:521–539, November 2011.

[17] Borja-Tabora C. F. C, Montalban C, Memish Z. A, et al. Long-term immunogenicity and safety after a single dose of the quadrivalent meningococcal serogroups A, C, W, and Y tetanus toxoid conjugate vaccine in adolescents and adults: 5-year follow-up of an open, randomized trial. *BMC infectious diseases*, 15:409, October 2015.

[18] World Health Organization . Who vaccine-preventable diseases: monitoring system. 2020 global summary. `https://apps.who.int/immunization_monitoring/globalsummary`, 2019. Accessed: 2020-07-09.

[19] KSA Ministry of Health . Statistical yearbook 2018. `https://www.moh.gov.sa/en/Ministry/Statistics/book/Documents/book-Statistics.pdf`, 2018. Accessed: 2020-07-09.

[20] World Health Organization . Saudi arabia: Who and unicef estimates of immunization coverage: 2018 revision. `https://www.who.int/immunization/monitoring_surveillance/data/sau.pdf`, 2018. Accessed: 2020-07-09.

[21] Memish Z, Al Hakeem R, Al Neel O, Danis K, Jasir A, and Eibach D. Laboratory-confirmed invasive meningococcal disease: effect of the Hajj vaccination policy, Saudi Arabia, 1995 to 2011. *Euro surveillance : bulletin Europeen sur les maladies transmissibles = European communicable disease bulletin*, 18, September 2013.

[22] Memish Z, Steffen R, White P, et al. Mass gatherings medicine: public health issues arising from mass gathering religious and sporting events. *Lancet (London, England)*, 393:2073–2084, May 2019.

[23] Yezli S, Assiri A. M, Alhakeem R. F, Turkistani A. M, and Alotaibi B. Meningococcal disease during the hajj and umrah mass gatherings. *International journal of infectious diseases : IJID : official publication of the International Society for Infectious Diseases*, 47:60–64, June 2016.
